# Design, Synthesis, and Drilling Fluid Performance of a Non-Organosilicon-Fluorine, High-Temperature, Comb-Shaped Zwitterionic Polymer Viscosity Reducer

**DOI:** 10.3390/molecules31142407

**Published:** 2026-07-08

**Authors:** Junxiong Zhao, Juanping Zhang, Shengchao Xu, Leilei Wang, Xiaochen Li, Yiping Chen, Yan Yang, Guangming Xu

**Affiliations:** 1 School of Technology for Sustainability, Beijing Normal University, Zhuhai 519087, China; 2 School of Chemistry and Chemical Engineering, Harbin Institute of Technology, Harbin 150001, China; 3 CNPC Bohai Drilling Engineering Company Limited, Tianjin 300280, China; 4 Research and Development Center, Yunnan Tin New Material Company Limited, Kunming 650200, China; 5 Faculty of Arts and Sciences, Beijing Normal University, Zhuhai 519087, China

**Keywords:** drilling fluid, viscosity reducer, high-temperature resistance, environmentally oriented, comb-type polymer

## Abstract

To address the potential ecological risks and environmental persistence of organosilicon-fluorine viscosity reducers in conventional silicone-fluoride drilling fluid systems, this work designs and synthesizes a non-organosilicon-fluorine, high-temperature, comb-shaped zwitterionic polymer viscosity reducer, AD-XSJ. The viscosity reducer is prepared via aqueous free-radical polymerization of acrylic acid (AA), acrylamide (AM), 2-acrylamido-2-methylpropane sulfonic acid (AMPS), and dimethyl diallyl ammonium chloride (DADMAC), and it exhibits low molecular weight, uniform molecular weight distribution, and excellent thermal stability. Analyses by FT-IR, thermogravimetry, particle size, zeta potential measurements and Electrostatic potential (ESP) demonstrate that AD-XSJ dismantles the bentonite network structure through the synergistic combination of hydrogen-bonding adsorption and electrostatic repulsion, releasing trapped free water and thereby substantially reducing viscosity and gel strength. Compared with conventional organosilicon-fluorine viscosity reducers, AD-XSJ exhibits superior viscosity reduction capability under high-solid, high-temperature, and high-salinity calcium-contamination conditions, achieving viscosity reduction rates of 33.3% and 50.0% in fluids contaminated with 10.0% NaCl and 1.0% CaCl_2_, respectively. In field applications under conditions of high bentonite content and calcium contamination, the viscosity reduction rates reach 57.7% and 62.5%, accompanied by markedly improved rheological properties and an average borehole enlargement rate of only 5.7%, indicating effective shale inhibition and anti-sloughing performance. Integrating efficient viscosity reduction, dispersion stabilization, and inhibition capabilities, this viscosity reducer can replace traditional organosilicon-fluorine products, reduce potential hazards to aquatic ecosystems at the source, and holds considerable promise for engineering and environmentally conscious deployment.

## 1. Introduction

As global oil and gas exploration and development extend into deeper and more challenging formations, drilling fluids play a critical role in drilling operations, and their performance directly affects drilling efficiency and operational safety [1,2,3]. High-temperature viscosity reducers, as essential functional components of drilling fluids, effectively regulate rheological properties and prevent excessive thickening of the system; their intrinsic characteristics therefore exert a decisive influence on fluid stability and drilling efficiency [4,5]. The silicone–fluoride drilling fluid system is a type of water-based drilling fluid that relies on an organosilicon-fluorine high-temperature viscosity reducer as the core treatment agent. Owing to its excellent thermal stability, shale swelling inhibition capacity, and lubricating properties, this system has been widely applied in recent years in complex well conditions such as deep wells and shale gas horizontal wells [6,7].

With increasing environmental awareness and progressively stricter regulatory oversight, however, the environmental impact of silicone–fluoride drilling fluid systems, especially their potential to contaminate aquatic environments, has attracted growing concern [8,9]. The organofluorine and organosilicon additives contained in such systems can still enter water bodies through multiple pathways, including the discharge of wastewater from material production, direct release and leakage of drilling fluids, and land disposal and leaching of waste drilling fluids, thereby posing various degrees of risk to surface water, groundwater, and aquatic ecosystems [10]. Recent studies indicate that organosilicon-fluorine compounds can disrupt the endocrine systems of aquatic organisms, affecting hormone levels and metabolic functions [11]; siloxanes exhibit slow metabolic rates in vivo, with half-lives ranging from weeks to months [8] and far exceeding those of most organic pollutants; and organofluorine compounds possess strong mobility and weak adsorption in the environment, making them prone to long-term accumulation in groundwater systems and the formation of persistent contamination [12,13].

It is therefore urgent to design, develop, and promote the field application of a novel, low-toxicity, readily degradable, non-organosilicon-fluorine high-temperature viscosity reducer substitute, so as to reduce the generation of organosilicon-fluorine pollutants at the source and meet both operational demands and regulatory requirements. Previous studies have found that water-soluble polymeric materials synthesized through free-radical polymerization of small organic molecules bearing carboxylic acid, amide, and sulfonic acid groups suitable for controlled polymerization exhibit favorable biodegradability under appropriate conditions, with complete mineralization yielding harmless small molecules such as CO_2_, H_2_O, and SO_2_ that cause no significant negative impact on aquatic ecosystems [14,15,16].

Moreover, the synergistic effect of sulfonate and carboxylate groups in the copolymer molecular structure imparts high calcium tolerance and salt resistance, allowing the molecular chains to remain extended under high-salinity and high-temperature conditions and thereby preventing salting-out and thermal degradation; the flexibility of the molecular chains and hydrogen bonding also enhance the adsorption and bridging capacity of the polymer [17,18]. Recent studies on zwitterionic polymer additives have shown that the introduction of both anionic and cationic groups, together with strongly hydratable sulfonate/amide moieties and rigid or sterically hindered units, can significantly improve the temperature, salt, and calcium tolerance of water-based drilling fluids. These polymers can adsorb firmly onto bentonite particles through hydrogen bonding, electrostatic attraction, and ionic interactions, thereby strengthening the hydration layer, suppressing salt- or calcium-induced aggregation, and promoting the formation of dense filter cakes. As a result, zwitterionic polymer-treated drilling fluids can maintain favorable rheological behavior and filtration-control performance even after high-temperature aging in highly saline or calcium-contaminated environments [19,20,21]. With its unique molecular structure design, this kind of polymer shows excellent scale inhibition, dispersion and corrosion inhibition performance under complex water quality conditions of high pH, high alkalinity, high hardness and high salinity [22,23,24,25], which is expected to solve the rheological control problem of high-density water-based drilling fluids under high-temperature and high-salt conditions.

Building on the viscosity-reducing and drag-reducing effects arising from the enhanced steric hindrance among solid particles imparted by comb-shaped polymers, together with the anti-electrolyte effect of zwitterionic polymers, this work designs and synthesizes an environmentally oriented and field-validated alternative to organosilicon-fluorine viscosity reducers, AD-XSJ, and systematically investigates its structure, properties, and field application. Through characterization techniques including FT-IR, thermogravimetric analysis, particle size distribution measurement, zeta potential analysis, ESP, and sedimentation stability tests, the mechanisms of adsorption, dispersion, and electrostatic repulsion between AD-XSJ molecules and bentonite particles are thoroughly analyzed, and the viscosity-reducing mechanism of dismantling the gel network and releasing trapped free water is elucidated. Compared with conventional organosilicon viscosity reducers, AD-XSJ exhibits superior viscosity reduction capability and system stability in both laboratory tests and field applications under high-solid, high-temperature, and high-salinity calcium-contamination conditions, along with a certain capacity to inhibit shale hydration and dispersion. These attributes indicate a promising prospect for replacing traditional organosilicon–fluorine viscosity reducers and for regulating the rheology of drilling fluids in complex formations.

## 2. Results and Discussion

### 2.1. Characterization of AD-XSJ

**Figure 1:**
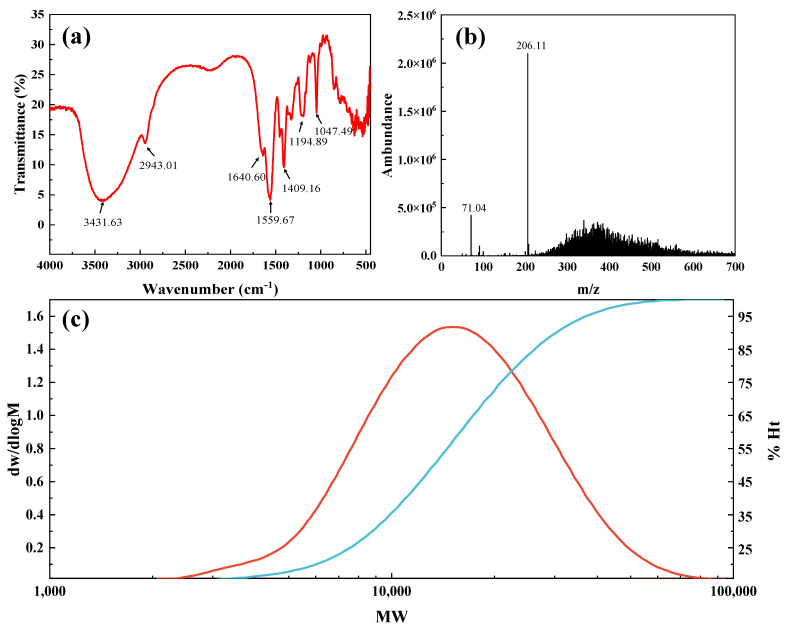
Characterization of the AD-XSJ viscosity reducer sample: (**a**) FT-IR spectrum; (**b**) negative-ion mass spectrum; (**c**) molecular weight and its distribution.

FT-IR spectroscopy, mass spectrometry, and molecular weight measurement are commonly employed to characterize the detailed structure of multi-monomer polymers [26,27,28]. The infrared spectrum of the purified and dried AD-XSJ product is presented in Figure 1a. The absorption band at 3431.63 cm^−1^ is assigned to the stretching vibrations of hydroxyl (–OH) and N–H bonds; the peak at 2943.01 cm^−1^ corresponds to the characteristic absorption of methyl (–CH_3_); the bands at 1640.60 cm^−1^ and 1559.67 cm^−1^ are attributed to the carbonyl stretching vibration of amide groups and the N–H bending vibration, respectively [29]; and the signals at 1194.89 cm^−1^ and 1047.49 cm^−1^ represent the characteristic absorptions of sulfonate (–SO_3_–) groups. These results confirm that the structure of AD-XSJ contains functional groups derived from all four monomers, consistent with the designed molecular architecture, and that the comb-shaped zwitterionic polymer AD-XSJ has been successfully prepared.

The negative-ion mass spectrum of the original AD-XSJ sample is shown in Figure 1b. The peak at *m*/*z* 71.04 corresponds to the characteristic fragment of the AA moiety, and the peak at *m*/*z* 206.11 corresponds to the AMPS moiety.

As shown in Figure 1c, the molecular weight of AD-XSJ is determined using an LC20 gel permeation chromatograph with an aqueous mobile phase. The weight-average molecular weight (*M*_w_) is 17,708 g/mol, the number-average molecular weight (*M*_n_) is 12,470 g/mol, and the polymer dispersity index (*M*_w_/*M*_n_) is 1.42. These results indicate that AD-XSJ is a low-molecular-weight polymer with a very uniform molecular weight distribution, meeting the molecular weight design criteria for an environmentally friendly high-performance viscosity reducer [30,31].

### 2.2. Characterization of the Viscosity-Reducing Performance of AD-XSJ and Mechanistic Explanation

To investigate the viscosity reduction performance of the synthesized comb-shaped zwitterionic polymer AD-XSJ, assess its potential for field application, and elucidate the underlying viscosity-reducing mechanism, thermogravimetric analysis, particle size measurement, sedimentation stability tests, and zeta potential analysis are conducted.

**Figure 2:**
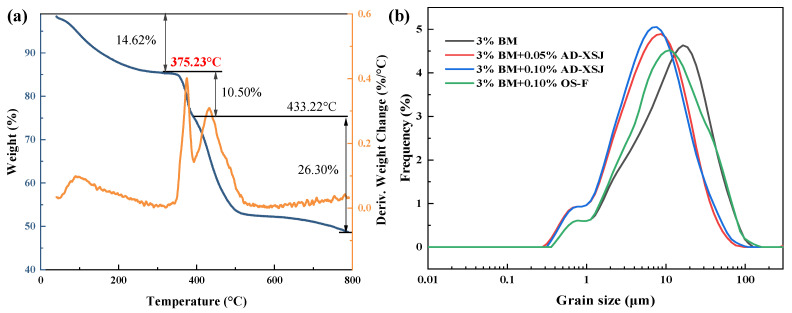
(**a**) TGA curve of the dried AD-XSJ sample (under nitrogen atmosphere); (**b**) comparison of particle size distributions of base slurries with different AD-XSJ and OS-F additions.

The thermal stability of AD-XSJ is evaluated by thermogravimetric analysis of the dried product after dialysis. The thermogravimetric (TG) curve, the core output of TGA, records the mass change in a substance as a function of temperature or time under programmed temperature control [32]. As shown in Figure 2a, below 375.23 °C the TG curve exhibits a gentle decline with slow mass loss, indicating the absence of significant thermal decomposition and demonstrating that the polymer can withstand a high-temperature environment up to 375.23 °C. Above 375.23 °C the curve drops sharply, reflecting pronounced thermal degradation, structural damage, and impaired performance. The mass retention of AD-XSJ at 375.23 °C remains above 85%, confirming its good thermal stability and suitability for the high-temperature downhole conditions encountered in most deep and ultra-deep wells.

Particle size distributions are measured using a laser particle size analyzer after homogenizing the test slurries. The frequency distribution curves are shown in Figure 2b. The base mud (BM) exhibits a monomodal distribution with a D_50_ of 13.9 μm. After adding 0.05% AD-XSJ, the D_50_ decreases to 7.32 μm; with 0.10% AD-XSJ, the D_50_ further decreases to 6.88 μm. In contrast, the D_50_ of the sample only decreased to 11.21 μm after adding 0.10% OS-F. These results indicate that AD-XSJ substantially reduces the particle size of the base mud, thereby hindering the formation of card-house structures through face-to-face and edge-to-face associations and dismantling the spatial network. In addition, AD-XSJ significantly reduces interparticle coalescence and frictional resistance through steric hindrance, thereby promoting dispersion.

**Figure 3:**
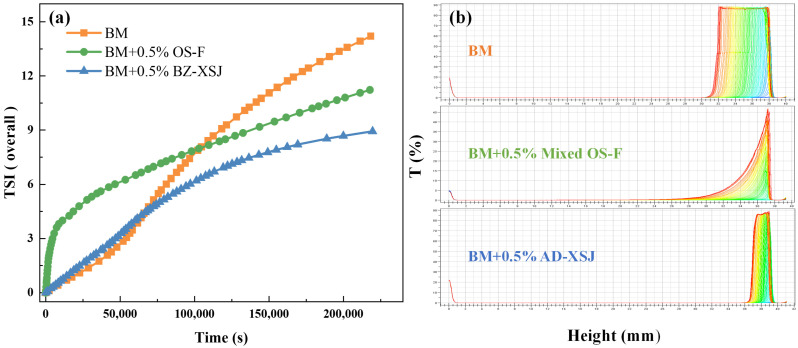
Sedimentation stability test of the base slurry and slurries with 0.5% OS-F and 0.5% AD-XSJ, respectively. (**a**) TSI value versus standing time curves; (**b**) variation curve of light transmittance intensity at different heights during sample standing.

Sedimentation stability is assessed using a stability analyzer after equilibrating the test slurries at 60 °C under static conditions. This instrument evaluates the physical stability of samples such as emulsions and suspensions by monitoring particle sedimentation, creaming, and phase separation, employing a pulsed near-infrared light source and measuring transmitted light intensity at multiple angles to quantify stability [33]. The Turbiscan Stability Index (TSI), calculated from Turbiscan multiple light scattering data, serves as an integrated stability parameter that reflects the sedimentation kinetics of opaque systems and the overall dynamic instability, with higher TSI values corresponding to greater instability [34]. As shown in Figure 3b, monitoring the transmitted light intensity at different heights of the sample vial over three days reveals that the base mud develops a pronounced plateau peak at 31–38 mm as the transmitted intensity increases with settling time (accompanying the transition from the green to the red spectrum). This feature is attributed to sediment accumulation at the bottom and clarification of the supernatant. By contrast, the samples containing 0.5% OS-F and 0.5% AD-XSJ both exhibit markedly improved resistance to stratification, and the base mud with 0.5% AD-XSJ shows superior stability compared with that containing 0.5% OS-F, as confirmed by both the peak positions in Figure 3b and the TSI evolution in Figure 3a. These findings further demonstrate that AD-XSJ effectively promotes dispersion and enhances drilling fluid stability.

To further evaluate the effect of AD-XSJ on the coalescence stability of bentonite particles, zeta potential measurements are performed. A higher absolute zeta potential indicates stronger interparticle repulsion and better dispersion and coalescence stability [35]. The zeta potential of the base evaluation slurry was −28.3 mV. After adding 0.4% AD-XSJ and 0.4% OS-F, respectively, the potentials correspondingly changed to −41.8 mV and −42.3 mV, indicating that this viscosity reducer has a dispersing ability comparable to that of traditional organic silicon–fluorine viscosity reducers, significantly enhancing the electrostatic repulsion between bentonite particles and improving the coalescence stability of the system.

**Figure 4:**
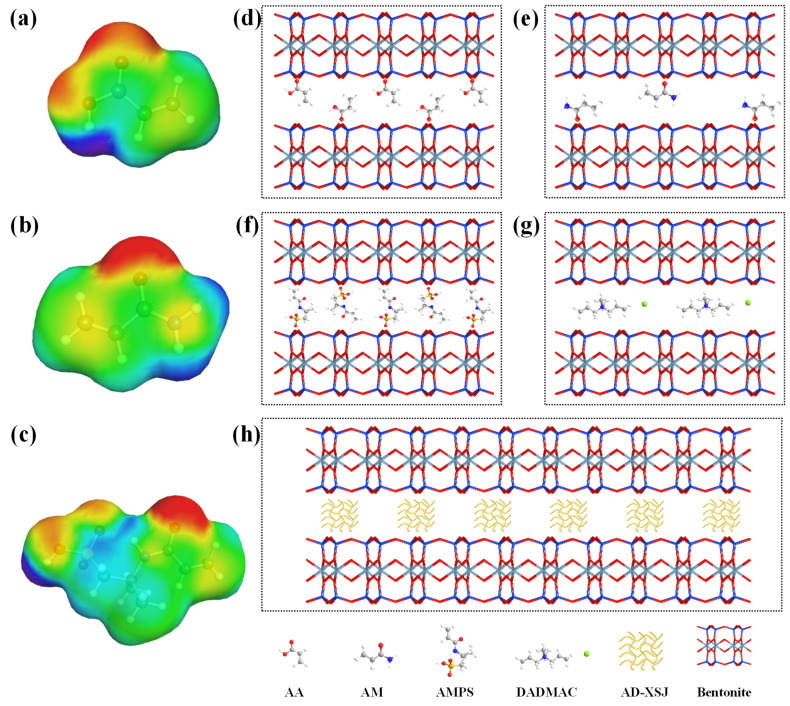
Surface electrostatic potential maps of the monomers used for synthesizing AD-XSJ and schematic diagrams of their interactions with bentonite particles: (**a**) surface electrostatic potential map of AA; (**b**) surface electrostatic potential map of AM; (**c**) surface electrostatic potential map of AMPS; (**d**) interaction between AA and bentonite; (**e**) interaction between AM and bentonite; (**f**) interaction between AMPS and bentonite; (**g**) interaction between DADMAC and bentonite; (**h**) interaction between AD-XSJ and bentonite.

Electrostatic potential (ESP) analysis is an effective computational tool for obtaining key information on the properties of target molecules [36,37]. As shown in Figure 4a–c, the ESP-mapped van der Waals surfaces of AA, AM, and AMPS are computed and visualized using the Multiwfn and VMD programs, where blue and red regions correspond to the maxima and minima of the electrostatic potential on the surface, respectively. Taking the amide group as an example, the nitrogen atom in amide adopts sp^3^ hybrid orbitals for bonding, and the hybrid orbital accommodating the lone pair on nitrogen possesses a higher p-orbital character. This lone pair conjugates with the π electrons of the C=O group through p-π conjugation, thereby increasing the electron density on the oxygen atom of the carbonyl. Consequently, the oxygen atom exhibits the most negative electrostatic potential in the entire molecule and is highly likely to serve as the active center for electrophilic reactions [38]. Therefore, AA, AM, and AMPS can stably bind to bentonite particles through hydrogen bonding and other interactions, which is directly corroborated by the FT-IR results presented in the preceding section.

Taken together, the above analyses indicate that the comb-shaped zwitterionic polymer AD-XSJ contains carboxylic acid, amide, thermally stable sulfonate, and inhibitive cationic groups in its molecular structure, with a relative molecular mass of approximately 17,000. Its small molecular size allows preferential adsorption onto bentonite particle surfaces through hydrogen bonding, thereby displacing pre-adsorbed high-molecular-weight polymers and disrupting the bridging network structure formed between polymers and bentonite [39], as illustrated in Figure 4d–h. AD-XSJ can also complex with macromolecular polymers in the system to form a cross-linked architecture, further impeding the structuring interactions between polymers and bentonite and effectively regulating the viscosity and gel strength of the drilling fluid. In addition, the anionic groups of AD-XSJ significantly increase the negative charge density on bentonite particle surfaces, thicken the hydration layer, and enhance electrostatic repulsion between particles [39,40].

**Figure 5:**
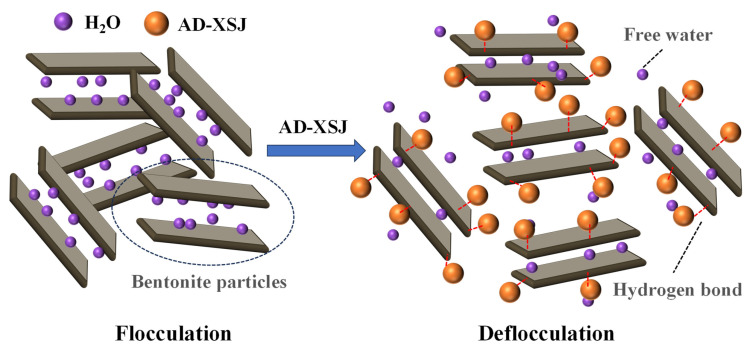
Schematic diagram of the interaction mechanism between viscosity reducer AD-XSJ and bentonite.

This not only weakens the three-dimensional network formed by edge-to-face or edge-to-edge associations of bentonite particles but also releases trapped free water, thereby reducing the structural viscosity (Figure 5). Finally, the small number of cationic groups introduced into AD-XSJ molecules can strongly adsorb onto bentonite surfaces through ion exchange, exhibiting strong adsorption affinity and persistence, which imparts a certain inhibitory effect on the hydration dispersion behavior of bentonite [41,42].

### 2.3. Performance Comparison Between AD-XSJ and OS-F

During drilling fluid service, elevated solid content, salt or calcium intrusion, and rising downhole temperatures intensify the interactions between macromolecular polymers and bentonite particles, leading to the formation of a denser network structure and consequently a pronounced increase in viscosity and gel strength [43]. It is therefore necessary to systematically evaluate and compare the performance of viscosity reducers under complex conditions involving high solid content, salt contamination, calcium contamination, and high temperature.

**Figure 6:**
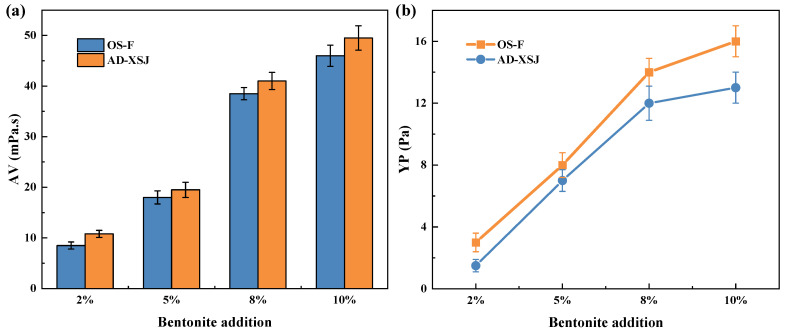
Changes in (**a**) apparent viscosity and (**b**) yield point of AD-XSJ and OS-F under different bentonite loadings.

To compare the viscosity-reducing effectiveness of AD-XSJ and OS-F in high-solid drilling fluids, the effect of bentonite loading on the rheological parameters of the evaluation slurries is determined at the same dosage (0.3%). As shown in Figure 6a,b, the apparent viscosity and gel strength of both viscosity reducer systems exhibit a clear upward trend with increasing bentonite content, which is primarily attributed to the higher degree of bentonite dispersion and the reinforcement of the network structure. At any given bentonite loading, the AD-XSJ system consistently displays lower viscosity and gel strength values, indicating its superior viscosity and gel reduction capability as well as its stronger resistance to solid-phase intrusion. This characteristic makes AD-XSJ particularly promising for application in formations with severe mud-making tendencies during the second spud section, such as the Minghuazhen Formation.

**Figure 7:**
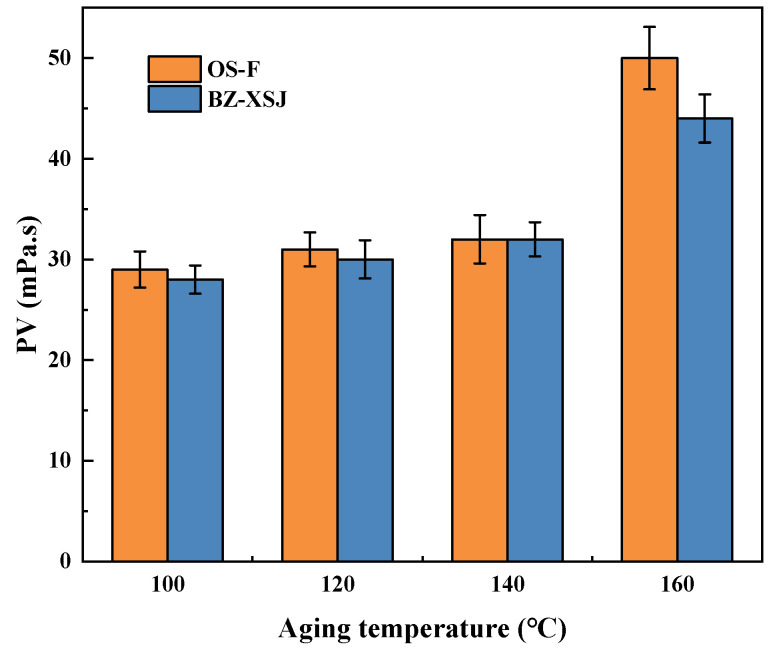
Changes in plastic viscosity of AD-XSJ and OS-F under different aging temperatures.

The thermal stability of the two viscosity reducers is compared using a base mud with high solid content (spiked with an additional 5% bentonite). As illustrated in Figure 7, the coalescence of bentonite particles intensifies with rising temperature, causing a significant increase in the apparent viscosity and gel strength of both systems. Under aging at 160 °C, however, the viscosity increase in the AD-XSJ system is markedly smaller than that of the OS-F system, demonstrating its superior thermal resistance. This behavior may be related to the incorporation of thermally stable functional groups such as sulfonate groups into the molecular structure, which help maintain polymer stability and sustained performance at elevated temperatures.

The viscosity reduction performance of the AD-XSJ and OS-F systems under salt contamination is further compared using the polymer-based drilling fluid from the second spud section of Well Nxx XX-X-XX as the base sample, in which the bentonite content is measured to be 156.75 g/L. The effects of contamination with NaCl and CaCl_2_ at different concentrations are examined and the results are summarized in Table 1. As the loading of NaCl and CaCl_2_ increases, the viscosity and gel reduction capabilities of both systems show a declining trend. This is mainly because salt intrusion compresses the diffuse double layer of bentonite particles, reduces the thickness of the hydration film, strengthens interparticle interactions, and ultimately forms a more compact network structure, which is manifested by elevated viscosity and gel strength. The AD-XSJ system maintains a viscosity reduction rate above 33% across all tests; at 10.0% NaCl, it achieves a viscosity reduction rate of 33.3%, and at 1.0% CaCl_2_, the rate reaches 50.0%. These results indicate that AD-XSJ possesses adequate resistance to salt and calcium contamination even in a high-solid environment, offering the potential to handle complex salt intrusion problems when drilling through salt-gypsum layers in formations with severe mud-making tendencies and to ensure stable drilling fluid properties. In comparison, the OS-F system exhibits significantly lower viscosity reduction efficiency, which may be ascribed to the specific functional groups built into the AD-XSJ molecule that enhance the steric hindrance among bentonite particles, promote particle dispersion, and thereby effectively mitigate flocculation and coalescence under high-solid and high-salinity conditions, improving the overall system stability.

Taken together, under identical high-solid evaluation conditions, AD-XSJ outperforms the OS-F system in terms of resistance to solid-phase intrusion, salt contamination, calcium contamination, and thermal degradation. AD-XSJ therefore demonstrates the capability to replace organosilicon-based viscosity reducers and is especially suitable for rheology control in complex formations and under extreme drilling fluid conditions, holding considerable promise for field application.

### 2.4. Effect of AD-XSJ Dosage on Viscosity Reduction Performance

To investigate the viscosity reduction performance of AD-XSJ under high-solid conditions, a high-solid evaluation slurry spiked with an additional 10% bentonite is used to examine the influence of different dosages on rheological parameters. As shown in Table 2, with increasing AD-XSJ dosage, the apparent viscosity, plastic viscosity, and yield point of the system exhibit a marked decreasing trend, while the viscosity reduction rate first increases and then decreases slightly. At a AD-XSJ dosage of 0.4%, the viscosity and gel strength of the system reach their lowest values and the viscosity reduction rate attains 70.7%, demonstrating that AD-XSJ retains excellent viscosity reduction capacity even in a high-solid environment. When the dosage exceeds 0.4%, the viscosity reduction effect declines to some extent, a phenomenon that may be associated with increased intermolecular association or secondary structuring among zwitterionic polymer chains. Therefore, the optimal AD-XSJ dosage under the conditions of this evaluation slurry is 0.4%.

### 2.5. Field Application of AD-XSJ in Wells with High Bentonite Content and Calcium Contamination

Several offset wells adjacent to Well Xxx XX employ a polymer system containing modified alkylsilane in the second spud section, while the offset wells of Well Fxxx XX-XX use a mosaic shielding system incorporating a combination of organosilicon and modified alkylsilane polymers. Conducting replacement trials of organosilicon–fluorine viscosity reducers in these two wells therefore offers considerable practical significance.

In Well Xxx XX, after drilling the cement plug, incomplete displacement of the drilling fluid results in a certain degree of calcium contamination, with a measured calcium ion concentration of 400 mg/L. In addition, in the highly mud-making Minghuazhen Formation of the second spud section, the bentonite content of the drilling fluid is allowed to rise without artificial intervention; once the bottom boundary of this formation is approached, the viscosity reducer (AD-XSJ) is added to evaluate its viscosity reduction performance under the harsh field conditions of high bentonite content and calcium contamination.

**Figure 8:**
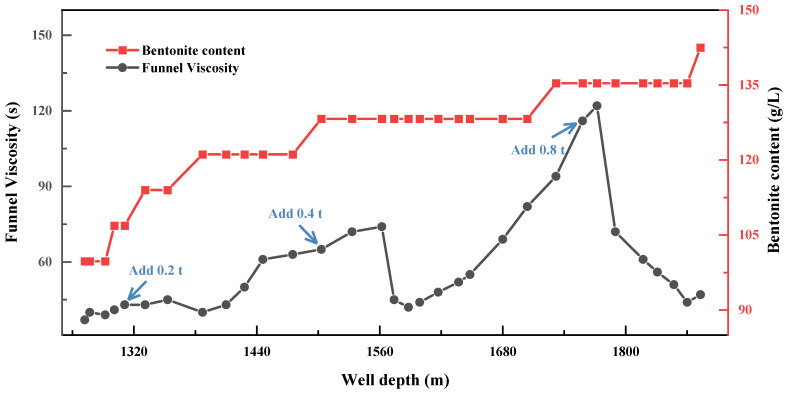
Variations in funnel viscosity and bentonite content of the drilling fluid with drilling footage in Well Xin-XX.

As shown in Figure 8, in the interval from 1272 to 1873 m, drilling fluid samples are collected at the shale shaker outlet every half hour to measure funnel viscosity and bentonite content. During the initial stage before AD-XSJ addition, the bentonite content rises rapidly to 106.88 g/L. Upon entering the Guantao Formation, further filtration control and a gradual increase in mud weight become necessary, and the addition of filtration-control polymers, barite, and other materials causes the viscosity and gel strength of the drilling fluid to rise continuously.

After two initial additions of 0.2 t AD-XSJ on each occasion, the viscosity and gel strength of the drilling fluid are kept under stable control. As the rate of penetration accelerates and more filtration-control polymers, barite, hydrolyzed ammonium polyacrylonitrile, modified plant fibers, and other materials are introduced, the funnel viscosity peaks at 122 s at a well depth of 1772 m. Subsequently, the dosage of AD-XSJ is increased, and the viscosity and gel strength are further controlled, with the funnel viscosity stabilizing at approximately 42 s.

Furthermore, as presented in Table 3, recording from the moment of maximum funnel viscosity shows that after the addition of AD-XSJ all rheological parameters decrease significantly. Within approximately three circulation cycles, the viscosity reduction rate reaches 57.7%, the funnel viscosity drops by 63.9%, the yield point decreases by 77.4%, the φ6 and φ3 readings decline markedly, and the initial and final gel strengths return to an appropriate level. These results demonstrate that AD-XSJ possesses outstanding viscosity and gel reduction capability even under the conditions of high bentonite content and cement calcium contamination encountered in Well Xxx XX.

Finally, from the initiation of viscosity reducer addition to the end of the second spud section, the average borehole enlargement rate over this interval is 5.7%. This indicates that the polymer drilling fluid containing AD-XSJ provides strong inhibition and improved wellbore stability.

In the trial at Well Fxxx XX-XX, a gypsum layer approximately 30 m thick is penetrated near the top of the Kong-1 Formation (at around 1500 m). Moreover, as in Well Xxx XX, the bentonite content in the highly mud-making Minghuazhen Formation at the beginning of the second spud section is left uncontrolled; at a well depth of 1561 m, the measured bentonite content reaches 163.875 g/L.

As shown in Table 4, AD-XSJ is added after drilling through the gypsum layer. After three circulation cycles, the viscosity reduction rate attains 62.5%, the funnel viscosity decreases by 69.9%, the yield point drops by 71.9%, the φ6 and φ3 readings decline markedly, and the initial and final gel strengths return to normal. These results also confirm that AD-XSJ exhibits excellent viscosity reduction performance under conditions of high bentonite content and calcium contamination from gypsum layers in Well Fxxx XX-XX.

## 3. Experimentation

### 3.1. Materials

Acrylic acid (AA, 99%), acrylamide (AM, 99%), 2-acrylamido-2-methylpropane sulfonic acid (AMPS, 98%), dimethyldiallylammonium chloride (DADMAC, 60% in water), ammonium persulfate (APS, ≥96%), sodium thiosulfate (≥97%), and sodium hydroxide (≥98%) were all purchased from Shanghai Aladdin Biochemical Technology Co., Ltd. (Shanghai, China) Deionized water was prepared in the laboratory.

### 3.2. Preparation of AD-XSJ

A comb-type zwitterionic polymer viscosity reducer, designated as AD-XSJ, was synthesized via free-radical polymerization in aqueous solution. The specific synthesis procedure is as follows: AA, AM, AMPS, and DADMAC were dissolved in deionized water at a molar ratio of 4:1.5:1:0.1 and stirred until fully homogeneous. The total monomer concentration was 40 wt%, the reactor volume was 5 L, and the stirring rate was 60 rpm. The mixture was heated to 60–70 °C under nitrogen protection during the heating process, after which a small amount of initiator (a mixed solution of 20 wt% ammonium persulfate and 20 wt% sodium thiosulfate) was introduced. The reaction was allowed to proceed for 5 h. Finally, the pH value was adjusted to 10–12 with sodium hydroxide, yielding a light yellow, transparent, low-viscosity liquid.

### 3.3. Characterization and Testing Methods

The following instruments were used: a Nicolet 380 FT-IR spectrometer (Thermo Fisher Scientific, Madison, WI, USA), a Q Exactive hybrid quadrupole-Orbitrap mass spectrometer (Thermo Fisher Scientific, Waltham, MA, USA), an LC20 gel permeation chromatograph (Shimadzu Corporation, Kyoto, Japan), a Q600 SDT simultaneous thermogravimetric–differential thermal analyzer (TA Instruments, New Castle, DE, USA), a Malvern Mastersizer 3000 laser particle size analyzer (Malvern Panalytical, Malvern, UK), a TURBISCAN near-infrared stability analyzer (Formulaction, Castanet-Tolosan, France), a DT300 Zeta potential analyzer (Occhio Instruments, Beijing, China), a WT-2000C variable-frequency high-speed stirrer (Beijing Institute of Exploration Engineering, Beijing, China), an HTD-GL8 high-temperature roller heating oven, and an HTD-6ST six-speed rotational viscometer (Qingdao Hengtaida Electromechanical Equipment Co., Ltd., Qingdao, China).

The FT-IR spectrometer of AD-XSJ was recorded on a Nicolet 380 FT-IR spectrometer (Thermo Electron Corporation, Waltham, MA, USA). The powder sample was pressed into a pellet, and the spectrum was collected over the range of 400–4000 cm^−1^.

Particle size distribution and the median particle size (D_50_) were determined using a Malvern Mastersizer 3000 laser particle size analyzer or an equivalent instrument. Distilled water was used as the dispersant. The particle refractive index was set to 1.560, the absorption index to 0.01, and the scattering model was Mie. After the measurement, the minimum and maximum particle sizes were taken as the particle size range of the sample, and the particle size corresponding to 50% of the cumulative volume distribution was taken as D_50_.

Stability tests were conducted on the Malvern Mastersizer 3000 laser particle size analyzer. The light source wavelength was 880 nm, the measurement temperature of the stability analyzer was 60 °C, the particle size measurement range was 1 nm to 100 mm, and the volume of the measurement cell was 20 mL.

The rheological properties of the sample were measured using an HTD-6ST six-speed rotational viscometer at 3, 6, 100, 200, 300, and 600 rpm. The rheological parameters of the drilling fluid were determined by the following formulas [44].

(1) Apparent viscosity (AV)=0.5 φ600 (mPa·s)

(2) Plastic viscosity (PV)=φ600−φ300 (mPa·s)

(3) Yield point (YP)=AV−PV (Pa)

(4) $$10s\;Gel\;strength\;\left(Initial\;gel\;strength\right)=0.5\;{\varphi 3}_{10\;s}\;(Pa)$$

(5) $$10min\;Gel\;strength\;\left(Initial\;gel\;strength\right)=0.5\;{\varphi 3}_{10\;min}\;(Pa)$$

where *φ*3_10s_ is the maximum value of 3 rpm readings after 600 r/min rotating for 10 s and then stand still for 10 s, *φ*3_10min_ is the maximum value of 3 rpm readings after 600 r/min rotating for 10 s and then stand still for 10 min.

The viscosity reduction rate of the sample is calculated by Equation (6) as follows:

(6) $$Viscosity\;reduction\;rate\;(DI)=\frac{{\varphi 100}_{0}-\varphi 100}{{\varphi 100}_{0}}\times \;100\%$$

where *φ*100_0_ is the reading of the original sample at 100 rpm, and *φ*100 is the reading of the original sample after adding viscosity reducer. *DI* represents the viscosity reduction rate.

### 3.4. Base Mud and Field Drilling Fluid Formulations

The base mud (BM) is prepared according to the Q/SH 0049–2007 standard of PetroChina and the API specifications [44]. At room temperature, 4% bentonite, 0.3% NaOH, 1% hydrolyzed ammonium polyacrylonitrile (NH_4_-HPAN), and 1% polymer-modified asphalt are added to a high-speed stirring cup containing 400 mL of deionized water and stirred with an electric stirrer at 1000 r/min for 30 min. Barite is then introduced to attain a density of 1.30 g/cm^3^, and stirring continues until uniform dispersion is achieved. The resulting suspension is sealed and cured at 25 °C ± 1 °C for 24 h.

For laboratory tests and field applications, the organosilicon–fluorine (OS-F) reference system is prepared according to the field operation protocol by mixing an anti-sloughing agent (organosilicon–fluorine modified polymer) with a liquid viscosity reducer (modified alkylsilane compound) in equal proportions.

The polymer drilling fluid formulation for the second spud section of Well Xxx XX consists of 2–4% bentonite, 0.2–0.4% filtrate reducer HV-CMC, 0.5–0.8% hydrolyzed ammonium polyacrylonitrile NH_4_-HPAN, 0.3–0.5% polyanionic cellulose PAC-LV, 1–1.5% anti-sloughing plugging agent polymerized humic acid SN, 1–1.5% anti-sloughing plugging agent, 0.2–0.3% encapsulator acrylate copolymer, 1–3% liquid lubricant modified vegetable oil, 0.8–1.0% liquid viscosity reducer AD-XSJ, and caustic soda flakes.

In Well Fxxx XX-XX, the polymer system is employed in the second spud interval from 511 m to 1528 m and is subsequently converted to a mosaic shielding system from 1528 m to 3101 m. The polymer formulation is identical to that applied in Well Xxx XX. The mosaic shielding drilling fluid is prepared by adding the following additives to the preceding polymer mud: 0.2–0.3% encapsulator acrylate copolymer, 1.5–3% liquid viscosity reducer AD-XSJ, 1.5–2.5% anti-sloughing plugging agent polymerized humic acid SN, 1–2% anti-sloughing plugging agent, 1–1.5% polyanionic cellulose PAC-LV, 2–3% liquid lubricant modified vegetable oil, and caustic soda flakes.

## 4. Conclusions

This study synthesizes a non-organosilicon–fluorine, high-temperature comb-shaped zwitterionic polymer viscosity reducer, AD-XSJ, through the copolymerization of acrylic acid (AA), acrylamide (AM), 2-acrylamido-2-methylpropane sulfonic acid (AMPS), and dimethyl diallyl ammonium chloride (DADMAC); its environmental advantage is primarily based on eliminating organosilicon–fluorine structural units from the molecular design and reducing potential sources of persistent silicon/fluorine-containing pollutants. Additionally, this study systematically investigates the structure and properties of this product. Characterization by FT-IR, thermogravimetry, particle size measurement, ESP, and zeta potential measurement confirms that AD-XSJ possesses low molecular weight, uniform molecular weight distribution, and good thermal stability, with mass retention exceeding 85% up to 375 °C. Results from particle size distribution, zeta potential, and sedimentation stability tests demonstrate that AD-XSJ, through the synergistic combination of hydrogen-bonding adsorption and electrostatic repulsion, effectively dismantles the network structure between bentonite particles, releases trapped free water, and significantly reduces the viscosity and gel strength of the system. Compared with conventional organosilicon viscosity reducers (OS-F), AD-XSJ exhibits superior viscosity reduction capability and system stability under high-solid, high-temperature, and high-salinity calcium-contamination conditions, achieving viscosity reduction rates of 33.3% and 50.0% in fluids contaminated with 10.0% NaCl and 1.0% CaCl_2_, respectively. Field application results demonstrate that under conditions of high bentonite content and calcium contamination, AD-XSJ attains viscosity reduction rates of 57.7% and 62.5% in Well Xxx XX and Well Fxxx XX-XX, respectively; effectively controls rheological properties; and yields an average borehole enlargement rate of only 5.7%, reflecting favorable inhibition and anti-sloughing performance. In summary, by virtue of multiple synergistic mechanisms, AD-XSJ integrates efficient viscosity reduction, dispersion stabilization, and inhibition capabilities, and can replace traditional organosilicon viscosity reducers. While ensuring rheology control in high-temperature, high-salinity complex formations, it markedly reduces the potential hazard of drilling operations to surface water and groundwater ecosystems, thus offering broad prospects for engineering application under increasingly stringent environmental regulations.

## 5. Application Prospects

The novelty of the present work lies in the integrated design and validation of a non-organosilicon–fluorine AA/AM/AMPS/DADMAC comb-shaped zwitterionic polymer with low molecular weight, narrow dispersity, high thermal stability, salt/calcium tolerance, and field performance under high bentonite content and calcium-contaminated conditions. The successful development and field validation of AD-XSJ provide a viable technical pathway for reducing organosilicon–fluorine-containing pollutant emissions from drilling operations at the source.

From the perspectives of environmental protection and ecological safety, some compounds in traditional organosilicon–fluorine viscosity reducers are characterized by strong environmental persistence, long-range mobility, and high bioaccumulation potential, while siloxanes exhibit half-lives in organisms ranging from weeks to months, imposing long-term stress on aquatic ecosystems that cannot be ignored. By replacing silicon–fluorine functional groups with an all-organic molecular backbone, AD-XSJ eliminates the generation of silicon- and fluorine-containing pollutants at the molecular design stage and essentially removes the persistent chemical stress of drilling fluid discharge on surface water and groundwater ecosystems. Against the backdrop of increasingly stringent wastewater discharge standards and chemical environmental access regulations facing the global oil and gas industry, the wider application of such environmentally friendly viscosity reducers is expected to become a key technical pillar of the green transformation of drilling fluid systems, offering an effective pathway for reconciling oil and gas exploration and development with ecological and environmental protection.

In terms of engineering economics and social benefits, AD-XSJ demonstrated high viscosity reduction efficiency, with viscosity reduction rates reaching 57.7% and 62.5%, and favorable borehole stability, with an average borehole enlargement rate of only 5.7%, in two field trial wells, indicating that this viscosity reducer can significantly reduce the frequency of drilling fluid maintenance and the consumption of treatment chemicals while effectively minimizing downhole complications caused by wellbore instability, shortening non-productive time, and thereby lowering overall drilling costs. Moreover, as China advances its strategy of carbon peaking and carbon neutrality and intensifies the control of emerging contaminants, enterprises adopting environmentally friendly drilling fluid additives will gain distinct institutional advantages in terms of environmental compliance and ESG ratings, enhancing their social image and market competitiveness.

Driven by the dual forces of the green transition of the global oil and gas industry and increasingly strict environmental regulations, the broader application of such environmentally friendly drilling fluid additives is expected to significantly reduce the potential hazards of drilling operations to surface water and groundwater ecosystems and promote the coordinated development of oil and gas exploration and environmental protection.

Finally, because there are no biodegradation and ecotoxicity tests in this study, these tests should be included in the future environmental safety assessment before large-scale deployment.

## Figures and Tables

**Table 1:** Viscosity reduction rates of AD-XSJ and OS-F in different brines.

Viscosity Reducer	Pollutants	AV/mPa·s	PV/mPa·s	YP/Pa	φ_100_	DI/%
AD-XSJ	-	10.0	8.0	2.0	5.0	83.3
	1.0% NaCl	12.5	9.5	3.0	7.0	76.7
	2.0% NaCl	18.5	14.0	4.5	10.0	66.7
	5.0% NaCl	23.0	18.0	5.0	14.0	53.3
	10.0% NaCl	28.5	21.0	7.5	20.0	33.3
	0.5% CaCl_2_	11.0	9.0	2.0	6.0	80.0
	1.0% CaCl_2_	23.0	16.0	7.0	15.0	50.0
OS-F	-	12.0	9.5	2.5	7.0	76.7
	1.0% NaCl	15.5	12.0	3.5	9.0	70.0
	2.0% NaCl	23.0	17.0	6.0	14.0	53.3
	5.0% NaCl	29.0	21.0	8.0	17.0	43.3
	10.0% NaCl	33.0	24.0	9.0	23.0	23.3
	0.5%CaCl_2_	17.0	12.0	5.0	9.0	70.0
	1.0% CaCl_2_	30.0	22.0	8.0	20.0	33.3

**Note**: AD-XSJ and OS-F were added at a concentration of 1.0%. The experiment was repeated five times.

**Table 2:** Effect of AD-XSJ dosage on the rheological parameters of the evaluation slurry.

AD-XSJ/%	AV/mPa·s	PV/mPa·s	YP/Pa	φ_100_	DI/%
0	54.5	37.0	17.5	41.0	-
0.1	46.0	33.0	13.0	28.0	31.7
0.2	31.0	23.0	8.0	19.0	53.7
0.3	26.0	18.0	8.0	17.0	58.5
0.4	23.5	19.0	4.5	12.0	70.7
0.5	26.0	20.0	6.0	15.0	63.4

**Note**: The experiment was repeated five times.

**Table 3:** Viscosity-reducing effect of AD-XSJ in a high-bentonite-content drilling fluid contaminated with cement and calcium at the Xin-XX well site.

Well Depth/m	Funnel Viscosity/s	φ_100_	φ_6_	φ_3_	Gel		YP/Pa	DI/%
					10″	10′		
1772.0	122.0	26.0	20.0	18.0	9.0	26.0	15.5	-
1817.0	61.0	19.0	10.0	7.0	5.0	19.0	9.0	26.9
1847.0	51.0	13.0	8.0	5.0	4.0	16.0	5.0	50.0
1860.0	44.0	11.0	4.0	3.0	2.0	12.0	3.5	57.7

**Table 4:** Viscosity-reducing effect of AD-XSJ in a high-bentonite-content drilling fluid contaminated with calcium from gypsum layers at the Fxxx XX-XX well site.

Well Depth/m	Funnel Viscosity/s	φ_100_	φ_6_	φ_3_	Gel		YP/Pa	DI/%
					10″	10′		
1561.0	143.0	32.0	20.0	18.0	13.0	41.0	16.0	-
1575.0	104.0	21.0	11.0	7.0	8.0	30.0	10.0	34.4
1586.0	62.0	17.0	5.0	4.0	2.0	5.0	7.5	46.9
1602.0	43.0	12.0	2.0	1.0	0.5	3.0	4.5	62.5

## Data Availability

The data and materials used in this study are available upon request.

## Abbreviations

The following abbreviations are used in this manuscript:

AD-XSJ	The name code of the viscosity reducer synthesized in this study
AA	acrylic acid
AM	acrylamide
AMPS	2-acrylamido-2-methylpropane sulfonic acid
DADMAC	dimethyl diallyl ammonium chloride
FT-IR	Fourier Transform Infrared Spectroscopy
ESP	Electrostatic potential
APS	ammonium persulfate
D_50_	median particle size
AV	Apparent viscosity
PV	Plastic viscosity
YP	Yield point
DI	Viscosity reduction rate
*M*_w_	weight-average molecular weight
*M*_n_	number-average molecular weight
TG	thermogravimetric
TSI	Turbiscan Stability Index
OS-F	organosilicon–fluorine
BM	base mud
